# Characterization of MEDLE-1, a protein in early development of *Cryptosporidium parvum*

**DOI:** 10.1186/s13071-018-2889-2

**Published:** 2018-05-23

**Authors:** Jilan Fei, Haizhen Wu, Jiayuan Su, Chanchan Jin, Na Li, Yaqiong Guo, Yaoyu Feng, Lihua Xiao

**Affiliations:** 1 State Key Laboratory of Bioreactor Engineering, School of Resources and Environmental Engineering, East China University of Science and Technology, Shanghai, 200237 China; 2 School of Biotechnology, East China University of Science and Technology, Shanghai, 200237 China; 3 Key Laboratory of Zoonosis of Ministry of Agriculture, College of Veterinary Medicine, South China Agricultural University, Guangzhou, 510642 China

**Keywords:** *Cryptosporidium parvum*, MEDLE family, Invasion, Neutralization, Expression

## Abstract

**Background:**

*Cryptosporidium* spp. are important diarrhea-causing pathogens in humans and animals. Comparative genomic analysis indicated that *Cryptosporidium*-specific MEDLE family proteins may contribute to host adaptation of *Cryptosporidium* spp., and a recent study of one member of this family, CpMEDLE-2 encoded by *cgd5_4590*, has provided evidence supporting this hypothesis. In this study, another member of the protein family, CpMEDLE-1 of *Cryptosporidium parvum* encoded by *cgd5_4580*, which is distinct from CpMEDLE-2 and has no signature motif MEDLE, was cloned, expressed and characterized to understand its function.

**Methods:**

CpMEDLE-1 was expressed in *Escherichia coli* and polyclonal antibodies against the recombinant CpMEDLE-1 protein were prepared in rabbits. Quantitative PCR was used to analyze the expression profile of *cgd5_4580* in *C. parvum* culture. Immunofluorescence staining was used to locate CpMEDLE-1 expression in life-cycle stages, and *in vitro* neutralization assay with antibodies was adopted to assess the role of the protein in *C. parvum* invasion.

**Results:**

The results indicated that *cgd5_4580* had a peak expression at 2 h of *C. parvum* culture. CpMEDLE-1 was located in the mid-anterior region of sporozoites, probably within the dense granules. The neutralization efficiency of anti-CpMEDLE-1 antibodies was approximately 40%.

**Conclusions:**

The differences in protein and gene expression profiles between CpMEDLE-1 and CpMEDLE-2 suggest that MEDLE proteins have different subcellular locations, are developmentally regulated, could be potentially involved in the transcriptional regulation of the expression of parasite or host proteins and may exert their functions in different stages of the invasion and development process.

## Background

*Cryptosporidium* spp. are intestinal protozoa that have emerged as an important cause of diarrheal disease in both humans and animals [1]. The severity of *Cryptosporidium* infection depends on immune status of the host, varying from self-limiting diarrhea in immunocompetent individuals to chronic and life-threatening infection in immunocompromised patients [2]. No effective therapy and vaccines are available against these important pathogens.

Host adaptation is recognized as a general phenomenon in *Cryptosporidium* spp., with certain species associated with specific hosts [3]. For instance, *C. hominis* and *C. viatorum* are largely human-specific, and *C. andersoni* and *C. bovis* are mostly cattle-related. A few species such as *C. parvum* and *C. ubiquitum* have a broad host range and are generally associated with zoonotic transmission [4]. Even in the latter, host adaptation has been noticed at the subtype family level. For examples, among the three common subtype families in *C. parvum*, IIa normally infects cattle, IId is commonly seen in sheep and goats, while IIc is mostly restricted to humans [5].

Proteins involved in the initial interaction between parasites and hosts are considered candidates involved in host adaptation. A number of candidates are potentially involved in *Cryptosporidium* attachment and invasion of host cells, such as CP2, P23, gp900, gp15/45 and TRAP-C1 [6]. Most of these molecules are identified as surface or secreted proteins, which are usually encoded by subtelomeric genes or gene families [7]. Many of them are glycosylated and expressed following a unique schedule during intracellular development of the pathogen [8].

Whole genome sequencing of *C. parvum* has identified several subtelomeric *Cryptosporidium*-specific gene clusters, encoding putative secreted proteins [9]. The MEDLE family, named after its conserved sequence motif at the C-terminus, is one of them, encoded by two clusters of six genes in the 3' subtelomeric regions of chromosomes 5 and 6. Comparative genomics has identified the occurrence of only one copy of the gene in *C. hominis*, which differs from *C. parvum* MEDLE-3 gene at the nucleotide level by 13% [10]. A deletion of one copy of the MEDLE genes was also seen in the IId subtype family of *C. parvum*, which has a narrow host range compared with the IIa subtype family [11]. Therefore, it was suggested that MEDLE proteins may be involved in host adaptation of *Cryptosporidium* spp. Interestingly, *Cryptosporidium* species divergent from *C. parvum* and *C. hominis*, such as *C. muris* and *C. andersoni*, do not have any MEDLE genes [12].

In a previous study by our research group [13], we conducted preliminary characterization of CpMEDLE-2, a divergent member of the MEDLE family, and provided evidence to support the hypothesis that MEDLE proteins may play a potential role in the invasion of *C. parvum*. In order to gain more insight into the biological functions of MEDLE proteins, here we characterized CpMEDLE-1, which is the only one in the protein family of *C. parvum* that does not have the MEDLE motif at the C-terminus.

## Methods

### Parasite and cell culture

*Cryptosporidium parvum* oocysts (IOWA strain) were purchased from Waterborne, Inc. (New Orleans, LA, USA) and stored in antibiotics at 4 °C for less than two months after the harvest. Prior to use, oocysts were treated with 0.5% sodium hypochlorite on ice for 10 min and subsequently washed three times with phosphate-buffered saline (PBS) at 13,200× *g* for 3 min.

Human ileocecal adenocarcinoma HCT-8 cells (ATCC CCL-244) were obtained from the Chinese Academy of Sciences Shanghai Branch, and cultured in RPMI 1640 medium supplemented with 10% fetal bovine serum (FBS) and 1% penicillin-streptomycin solution (PS) at 37 °C under 5% CO_2_. For *in vitro* experiments, HCT-8 cells were seeded into 12-well plates with coverslips and allowed to grow onto coverslips overnight in 10% FBS-supplemented RPMI 1640 medium until reaching ~90% confluence. Afterwards, the culture medium was replaced with fresh 2% FBS-supplemented RPMI 1640 medium. Hypochlorite-treated oocysts were suspended in the culture medium and added into the plates at 5 × 10^5^ oocyst/well. Following a 2 h incubation for excystation and invasion, unexcysted oocysts and free sporozoites were removed by washes in PBS, with fresh 2% FBS-supplemented RPMI 1640 medium added to the culture. The cells were allowed to grow for specified time in different assays.

### Identification of MEDLE homologues and sequence analysis

The CpMEDLE-1 gene (*cgd5_4580*) was identified from the *C. parvum* IWOA genome sequences in the CryptoDB database (http://cryptodb.org) as an intronless gene. Amino acid sequences for other five MEDLE genes in *C. parvum* and the only MEDLE gene in the *C. hominis* TU502 genome were also retrieved from CryptoDB and aligned using ClustalX 2.0.11 [14]. Glycosylation sites were predicted using NetOlyc and NetNGlyc (https://www.expasy.org/glycomics). Potential antigenic epitopes were predicted by B Cell Epitopes Prediction Tools (http://tools.immuneepitope.org/main/bcell/). The phylogenetic relationship among MEDLE proteins was assessed by using the maximum likelihood method implemented in MEGA 7.0.26 [15], based on the Poisson distribution model. Bootstrap values were obtained by running 1,000 replicates.

### Cloning, expression and purification of CpMEDLE-1

The *cgd5_4580* gene was amplified from genomic DNA of the *C. parvum* IOWA isolate with the following primers (the added restriction sites are underlined): forward, 5'-AAA TCC ATG GAA AAT ATA ACC GAT AAT TT-3'; reverse, 5'-AAA TCT CGA GAC TTG TCT CTA CTT TTT TTT T-3'. The template DNA was extracted from *C. parvum* oocysts by using the Qiagen DNeasy Blood & Tissue Kit (Qiagen, Hilden, Germany). The PCR was performed in a GeneAmp 9700 (Applied Biosystems) with the following cycling conditions: 95 °C for 5 min; 35 cycles of 95 °C for 45 s, 50 °C for 45 s, and 72 °C for 1 min; and 72 °C for 10 min. The amplified product was purified using the SanPre PCR Product Purification Kit (Sangon Biotech, Shanghai, China) and cloned into an *Nco*I and *Xho*I double-digested pET28a vector. Recombinant plasmids generated were transformed into competent *Escherichia coli* DH5α cells. The bacterial colonies were screened using T7/T7t universal primers, with positive colonies being sequenced to confirm their identity and sequence accuracy.

The plasmid harboring the correct insert was transformed into *E. coli* BL21 (DE3) for protein expression. The BL21 (DE3) cells were cultured in LB medium containing 100 μg/ml kanamycin and grown at 37 °C until the OD_600_ reached 0.6–1.0, after which 0.5 mM IPTG was added to induce protein expression at 25 °C for 4 h. The expression level and solubility of the target protein were evaluated by using SDS-PAGE and western blot analysis. The band of the expected size was excised from the gel and further analyzed for identity by Matrix-Assisted Laser Desorption/Ionization Time of Flight Mass Spectrometry (MALDI-TOF-MS).

For protein purification, bacteria were inoculated into 2 l of fresh medium and cultured as described above. Afterwards, bacteria were collected by centrifugation, disrupted by sonication on ice and centrifuged again to remove cell debris. The supernatant was filtered through a 0.45 μm cellulose acetate membrane filter (Millipore, Billerica, MA, USA), and loaded onto Ni-NTA beads (Novagen, Madison, WI, USA) at 4 °C and 70 rpm for 2 h. After washing the beads with six volumes of 20 mM imidazole buffer, CpMEDLE-1 was eluted from the beads with elution buffers containing increasing concentrations of imidazole. The purified protein was concentrated by ultrafiltration with an Amicon® Ultra-15 10K Centrifugal Filter Devices (Millipore, Norcross, GA, USA), and examined on a 10% SDS-PAGE gel.

### Preparation of anti-CpMEDLE-1 antibodies

Polyclonal antibodies against CpMEDLE-1 were raised in pathogen-free rabbits by GI Biochem Ltd. (Shanghai, China). The primary immunization was conducted on days 1 and 15 using 350 μg of purified CpMEDLE-1 protein emulsified in an equal volume of Freund’s complete adjuvant. Immunized animals received boost immunizations six times every seven days with 150 μg of CpMEDLE-1 protein in Freund’s incomplete adjuvant. Seven days after the final immunization, rabbit sera were collected, and the polyclonal IgG antibodies were purified by using an affinity chromatographic column conjugated with CpMEDLE-1. The titer and specificity of the antibodies were evaluated using an enzyme-linked immunosorbent assay (ELISA) and western blot, respectively.

### Western blot analysis of native CpMEDLE-1

To assess the expression of CpMEDLE-1, free sporozoites were prepared from oocysts that were treated with 0.5% sodium hypochlorite as described above and incubated in PBS buffer containing 0.75% taurodeoxycholic acid and 0.25% trypsin at 37 °C for 1 h. The released sporozoites were collected, washed three times by centrifugation with PBS at 13,200× *g* for 3 min, and re-suspended in PBS containing 1% Triton X-100 and a protease inhibitor cocktail (Merck, Darmstadt, Germany). Proteins (from ~1 × 10^6^ sporozoites/lane) in the lysate were separated on a SDS-PAGE gel. After the transfer of proteins, the nitrocellulose membranes were blocked with 5% nonfat milk-PBST for 2 h, and incubated for 2 h in 5% nonfat milk-PBST supplemented with anti-CpMEDLE-1 antibodies (~0.4 μg/ml), post-immune serum (1:4000) or pre-immune serum (1:4000). Horseradish peroxidase (HRP)-conjugated goat-anti-rabbit antibodies (Yeasen, Shanghai, China) were employed at 1:5000 as the secondary antibodies in an incubation for another 1 h. The blots were washed three times with PBST after each incubation at room temperature (RT). The reactive protein bands were detected by using an enhanced chemiluminescent reagent (Thermo Fisher, Rockford, IL, USA).

### Assessment of cross-reactivity of anti-CpMEDLE-1 antibodies to other MEDLE proteins

To determine whether anti-CpMEDLE-1 antibodies cross-react with other MEDLE proteins, CpMEDLE-2 and two other MEDLE members under study in our research group, CpMEDLE-3 (encoded by *cgd5_4600*) and CpMEDLE-5 (encoded by *cgd6_5480*), were employed as antigens in incubations with anti-CpMEDLE-1 antibodies. The concentrations of individual proteins were determined by using a BCA Protein Quantification Kit (Yeasen, Shanghai, China). ELISA was performed to assess the dose response of anti-CpMEDLE-1 antibodies. For this, proteins were coated onto the ELISA plates and anti-CpMEDLE-1 antibodies (1.55 mg/ml) were added at dilutions of 1:1000, 1:2000, 1:4000, 1:8000 and 1:16,000. Pre-immune serum was employed as the negative control. HRP-conjugated goat-anti-rabbit secondary antibodies were used for detection of reactivity to the coated antigens by the anti-CpMEDLE-1 antibodies, with absorbance being measured at 450 nm. The relative absorbance values of samples to negative control greater than 2.1 were considered positive. The cross-reactivity of anti-CpMEDLE-1 antibodies to other MEDLE proteins was also assessed by using western blot analysis of MEDLE proteins (1 μg/lane) as described above.

### Quantitation of *cgd5_4580* gene expression

The relative expression level of the *cgd5_4580* gene in intracellular parasites in HCT-8 cultures at 0–72 h was evaluated by qPCR. The expression of *C. parvum 18S* rRNA (*Cp18S* rRNA) gene was used for data normalization. Total RNA at each culture point was isolated from *C. parvum*-infected HCT-8 cells using an RNeasy Mini Kit (Qiagen), and quantitated and assessed for purity using NanoDrop 2000. Each of the qPCR reaction contained 0.1 mM primers, 1 μl of cDNA synthesized from 2 μg of RNA using a GoScript™ Reverse Transcription System (Promega, Beijin, China), and 10 μl of SYBR Green PCR Mix (TOYOBO, Osaka, Japan) in a 20 μl volume. The reaction was run on a LightCycler**®** 480 (Roche, Basel, Switzerland), with 45 cycles of 95 °C for 30 s, 58 °C for 30 s, and 72 °C for 30 s. The following primers specific to *cgd5_4580* were used in qPCR: 5'-GGT TCG AGT AGA GGT GGA GGT-3' and 5'-AGA AGG GAC CAT AGC GAT CA-3' (amplicon size = 216 bp), together with published *Cp18S* rRNA qPCR primers 5'-CTC CAC CAA CTA AGA ACG GCC-3' and 5'-TAG AGA TTG GAG GTT GTT CCT-3' (amplicon size = 256 bp) [16]. The cultivation assays were carried out in triplicate and qPCR analysis of each RNA extraction was performed in duplicate. Relative levels of gene expression were calculated by using the 2^-△△CT^ method [17], where differences between threshold cycle (C_T_) values were first determined by computing the △C_T_ between target gene transcripts (C_T [CpMEDLE-1]_) and *Cp18S* rRNA (C_T[*Cp18S*]_) as △C_T_ = C_T [CpMEDLE-1]_ – C_T[*Cp18S*]_. The △C_T_ values of each time points were then normalized by subtracting the minimum △C_T_ value (△C_T[min]_) among them as △△C_T_ =△C_T_ – △C_T[min]_.

### Immunofluorescence assay (IFA)

Free sporozoites were harvested and re-suspended in a drop of PBS on a microscopy slide. Intracellular stages of *C. parvum* were obtained by infecting HCT-8 cells grown on coverslips for 24 and 48 h. The slide and coverslips were fixed at RT in methanol for 15 min. After three washes in PBS, the fixed cells were permeabilized with 0.5% Triton X-100 in PBS for 15 min, washed three times with PBS, blocked with 5% BSA in PBS (BSA-PBS) at RT for 1 h, and incubated with anti-CpMEDLE-1 antibodies (~ 0.4 μg/ml) in 5% BSA-PBS for 1 h. After three washes in PBS, the cells were incubated with Alexa Fluor® 594-conjugated Goat Anti-rabbit IgG (Cell Signaling Technology, MA, USA) in BSA-PBS at 1:400 for 1 h. After three washes with PBS, the cells were counter-stained with the nuclear stain 4′, 6-diamidino-2-phenylindole (DAPI, Roche, Basel, Switzerland). After another three washes with PBS, the slide or coverslips were mounted with No-Fade Mounting Medium (Booster, Wuhan, China) and examined by differential interference contrast (DIC) and fluorescence microscopy using a BX53 immunofluorescence microscope (Olympus, Tokyo, Japan).

### Invasion neutralization assay

Neutralization assays were performed to assess the potential role of CpMEDLE-1 protein in *C. parvum* invasion. Hypochlorite-treated oocysts were pre-incubated at 37 °C in medium containing increasing dilutions of post-immune serum for 15 min, with pre-immune serum or medium alone as controls. They were added onto HCT-8 cells grown to ~90% confluence in 12-well plates as described before. The dilutions of serum used included 1:200, 1:500 and 1:1000. After 2 h incubation, the HCT-8 cells were washed three times and incubated in fresh culture medium for additional 24 h. To assess the infection rate of HCT-8 cells, the culture on coverslips was stained with Cy3-labeled Sporo-Glo™ antibodies (Waterborne, New Orleans, LA, USA) and examined under the immunofluorescence microscope. Images were captured randomly from 50 microscope fields per coverslip under 200×, and the total number of parasites in the fields was quantified by using Image J 1.4.3.67 (https://imagej.nih.gov/ij/). All experiments were performed in triplicate and the data generated were compared using a Student’s t-test. The neutralization efficiency of anti-CpMEDLE-1 antibodies at different dilutions was calculated based on differences in parasite load between antibody-treated groups and their corresponding controls.

## Results

### Sequence characteristics of CpMEDLE-1

**Fig. 1:**
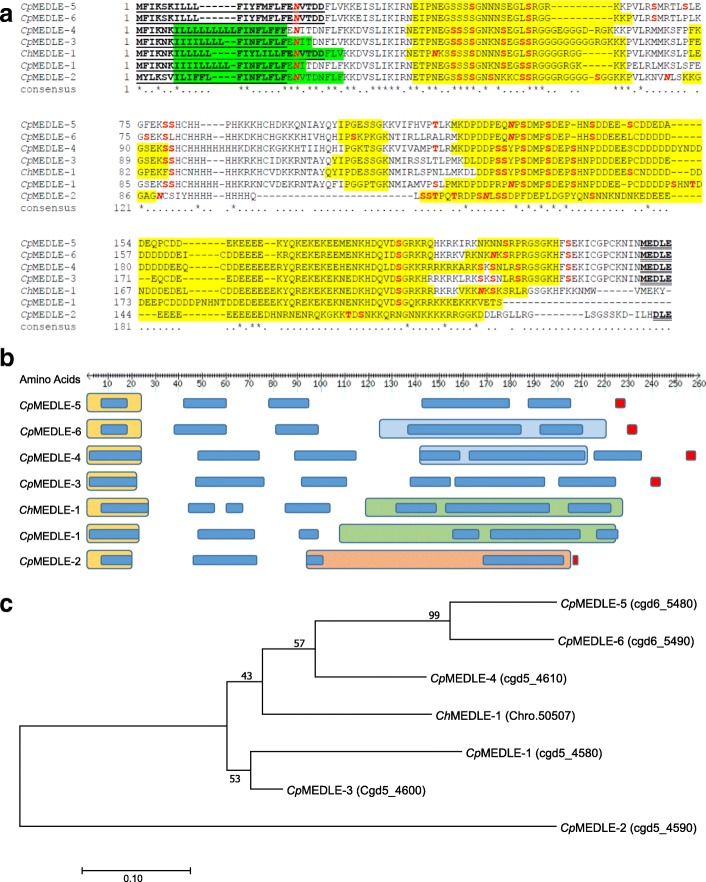
Sequence characteristics of MEDLE family proteins. **a** Multiple sequence alignment of MEDLE proteins in *C. parvum* and *C. hominis*. The *Cp* and *Ch* represent for *C. parvum* and *C. hominis*, respectively, and consensus sequence shows the amino acid identity with an asterisk and similarity with a period. The predicted signal peptide is shown in bold and underlined, the transmembrane domain is shaded in green, the predicted O-linked glycosylation sites are in red, while the N-linked glycosylation sites are in red and italicized. The predicted B cell epitopes of MEDLE proteins are shaded in yellow, and the motif “MEDLE” at the C-terminus is shown in bold and double underlined. **b** Motifs or domains of MEDLE proteins based on predictions in CryptoDB. The LCRs are shown in blue, the predicted signal peptide is shown in yellow, and the motif “MEDLE” is shown in red. The histone chaperone domain is shown in light blue, the ATP-dependent RNA helicase domain is shown in green, and the serine/threonine protein kinase domain is shown in orange. **c** Phylogenetic relationship of *C. parvum* and *C. hominis* MEDLE family proteins using MEGA 7.0.26. The tree was constructed by a maximum likelihood analysis based on evolutionary distances calculated using the position model. Bootstrap values were obtained by running 1000 pseudoreplicates

CpMEDLE-1 is a putative secretory protein encoded by the *cgd5_4580* gene in the 3' subtelomeric region of chromosomes 5 of *C. parvum*. The sequence contains 230 amino acids with a predicted signal peptide at amino acids 1–23 and a transmembrane domain at 7–26. It has 14 predicted O-linked glycosylation sites involving one threonine and 13 serine residues, as well as two predicted N-linked glycosylation sites at amino acids 25 and 143 (Fig. 1a). Among MEDLE proteins, by *in silico* analysis, there is an ATP-dependent RNA helicase domain in CpMEDLE-1 and ChMEDLE-1, a serine/threonine protein kinase domain in CpMEDLE-2, and a histone chaperone domain in CpMEDLE-4 and CpMEDLE-6. In addition, CpMEDLE-1 possesses six low complexity regions (LCRs) along the sequence, which are also present in other MEDLE proteins (Fig. 1b). Multiple sequence alignment of MEDLE family proteins of *C. parvum* and *C. hominis* had revealed a high sequence identity among them. However, CpMEDLE-1 appears to be a truncated member of the family and does not have the motif “MEDLE” at the C-terminus of the protein. CpMEDLE-2 is the most divergent member of the family, with only 36% sequence identity to CpMEDLE-1, compared with the 67% and 56% identities by CpMEDLE-3 and CpMEDLE-5, respectively. In B cell epitope prediction, the conserved regions of MEDLE proteins have overlapping epitopes, suggesting that polyclonal antibodies against CpMEDLE-1 could have immunological cross-reactivity with other MEDLE proteins. The phylogenetic tree inferred from *C. parvum* and *C. hominis* MEDLE proteins indicated that CpMEDLE-1 is most related to CpMEDLE-3, followed by ChMEDLE-1, CpMEDLE-4, CpMEDLE-5 and CpMEDLE-6 (Fig. 1c).

### Expression of recombinant CpMEDLE-1

**Fig. 2:**
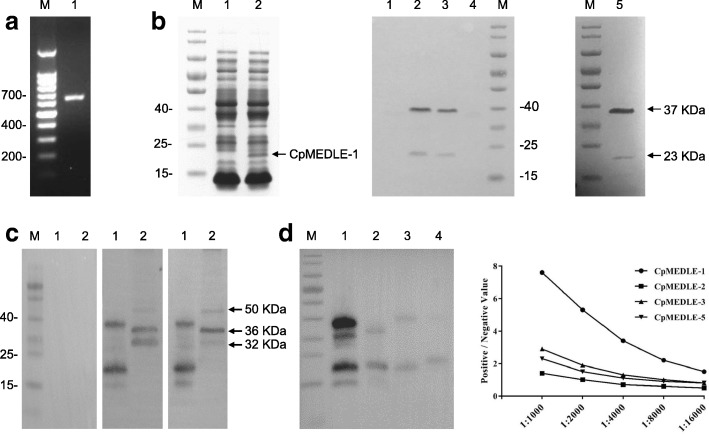
Production of recombinant CpMEDLE-1 and polyclonal antibody. **a** PCR amplification of the *cgd5_4580* gene of *C. parvum*. Lane M: 100 bp molecular makers; Lane 1: *cgd5_4580* PCR product. **b** Expression and purification of recombinant CpMEDLE-1. Recombinant CpMEDLE-1 protein expressed in *E. coli* BL21 (DE3) was analyzed by SDS-PAGE (left panel) and western blot (middle panel), while purified CpMEDLE-1 was analyzed by SDS-PAGE alone (right panel). Lane M: molecular weight makers; Lane 1: lysate from recombinant bacteria without IPTG induction; Lane 2: lysate from IPTG-induced recombinant bacteria, with the expected product indicated by an arrow; Lane 3: supernatant of IPTG-induced recombinant bacterial culture; Lane 4: sediment of lysate from IPTG-induced recombinant bacteria; Lane 5: CpMEDLE-1 purified using Ni-NAT affinity chromatography. **c** Expression of native MEDLE-1 protein in *C. parvum* sporozoites. Western blots were carried out using pre-immune serum (left panel), anti-CpMEDLE-1 antibodies (middle panel) and post-immune serum (right panel). Lane M: molecular weight makers; Lane 1: purified CpMEDLE-1 protein; Lane 2: crude protein extracted from sporozoites. **d** Cross-reactivity of anti-CpMEDLE-1 antibodies against other MEDLE proteins as revealed by western blot (left panel) and ELISA (right panel). In western blot analysis, 1 μg/lane of MEDLE proteins including CpMEDLE-1 (Lane 1), CpMEDLE-2 (Lane 2), CpMEDLE-3 (Lane 3) and CpMEDLE-5 (Lane 4) were co-incubated with anti-CpMEDLE-1 antibodies. In ELISA analysis, equal amounts of these four MEDLE proteins coated on plates were incubated with different dilutions of anti-CpMEDLE-1 antibodies, with pre-immune serum as the negative control. Any OD_450_ ratio greater than 2.1 was regarded as positive immunoreactivity

We cloned, expressed and purified recombinant CpMEDLE-1 protein with a His-tag (Fig. 2a, b). In western blot analysis, CpMEDLE-1 migrated with a molecular mass consistent with the predicted size of ~23 KDa. However, there was a consistent presence of another larger band of ~37 KDa. To confirm their CpMEDLE-1 identity, both bands were analyzed by using MALDI-TOF-MS, yielding peptide sequences of CpMEDLE-1 (Accession No. XM_625306; data not shown). Thus, the larger one could be a dimer of the CpMEDLE-1 protein. No obvious differences in the dimer formation were observed when treating the proteins with either dithiothreitol or ultracentrifugation.

For the characterization of CpMEDLE-1, we performed a series of western blots with the anti-CpMEDLE-1 antibody preparation and post-immune serum, with the pre-immune serum as control (Fig. 2c). The purified recombinant CpMEDLE-1 was recognized as expected, with two expected bands of ~23 KDa and ~37 KDa, and two other smaller bands that were more likely degraded products of CpMEDLE-1. In contrast, a total protein extract from sporozoites produced three bands of ~50 KDa, ~36 KDa and ~32 KDa, suggesting the possibility of antibodies reacting with multiple native MEDLE proteins in *C. parvum*. Because all three bands were larger than the predicted sizes of MEDLE proteins, post-translational O-linked and N-linked glycosylation could be responsible for the discrepancy in predicted and observed sizes of MEDLE proteins. As expected, the pre-immune serum had no reactivity with either purified CpMEDLE-1 or native protein.

### Cross-reactivity of CpMEDLE-1 antibodies with other MEDLE proteins

The cross-reactivity of anti-CpMEDLE-1 antibodies (1.55 mg/ml) against other MEDLE proteins was validated by western blot and ELISA (Fig. 2d). The result of western blot showed some cross-reactivity of anti-CpMEDLE-1 antibodies to CpMEDLE-2, CpMEDLE-3 and CpMEDLE-5, with relatively weak bands in blots of other MEDLE proteins compared with obvious recognition of CpMEDLE-1. The cross-reactivity of the antibodies was further evaluated by ELISA analysis using different dilutions of anti-CpMEDLE-1 antibodies, which showed a modest reactivity to CpMEDLE-3 and CpMEDLE-5 at 1:1,000 dilution. At this concentration, CpMEDLE-2 did not show any significant reactivity to anti-CpMEDLE-1 antibodies in ELISA. At the 1:4,000 and higher dilutions, the anti-CpMEDLE-1 antibodies had no significant reactivity to other MEDLE proteins in ELISA.

### Differential expression of CpMEDLE-1 in *C. parvum*

**Fig. 3:**
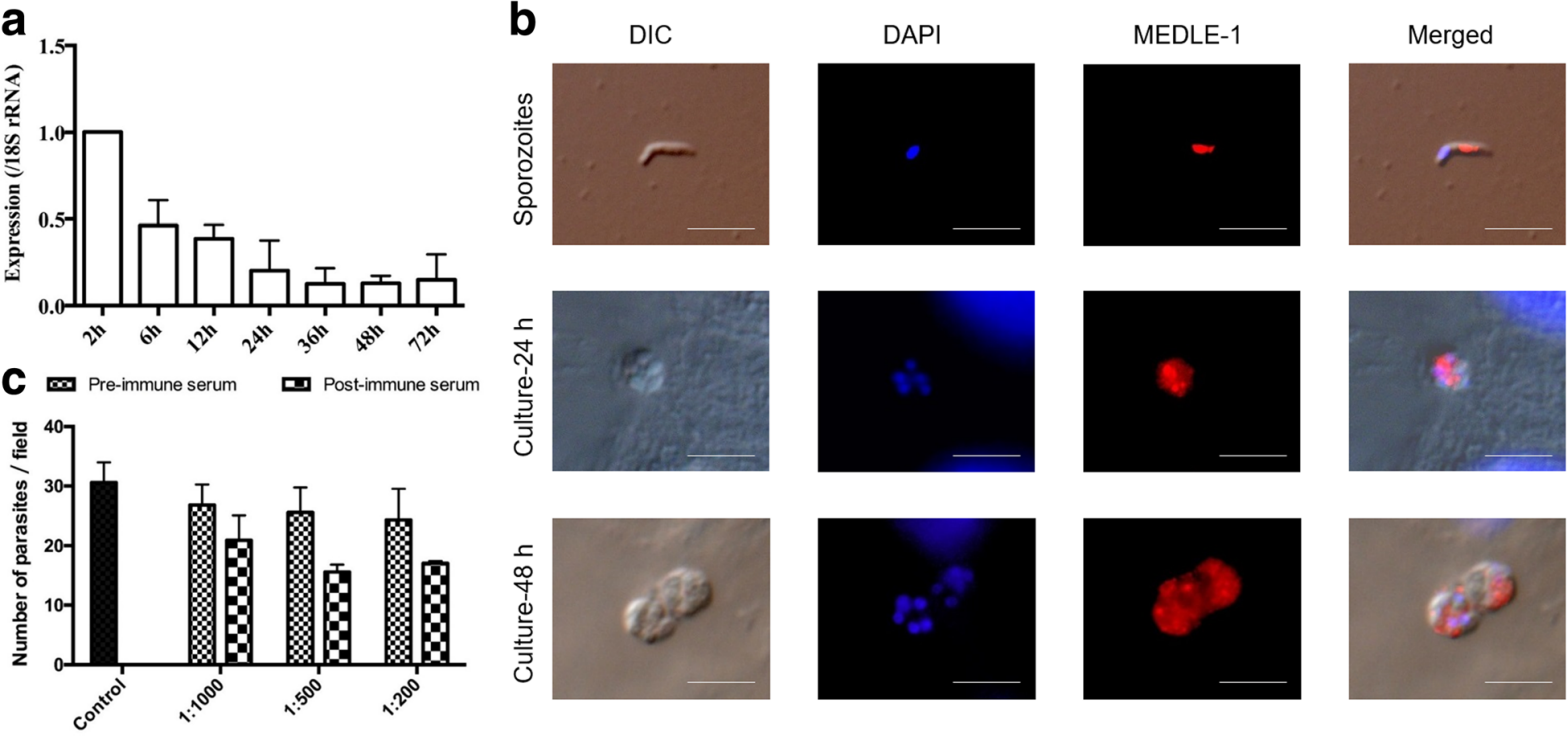
Biological characteristics of native CpMEDLE-1. **a** Relative expression levels of the *cgd5_4580* gene at various *C. parvum* culture time as determined by qPCR. Data from the *Cryptosporidium 18S* rRNA gene were used as an internal control for data normalization. Data presented are mean ± SD from three replicate cultivation assays, with qPCR analysis of each RNA extraction performed in duplicate. **b** Expression of CpMEDLE-1 on free sporozoites (top panel) and intracellular developmental stages of *C. parvum* in HCT-8 cell cultures at 24 h (middle panel) and 48 h (bottom panel). The images were taken under differential interference contrast (DIC), with nucleus counter-stained with 4', 6-diamidino-2-phenylindole (DAPI), parasites stained by immunofluorescence with Alexa 594-labled CpMEDLE-1 (MEDLE-1), and superimposition of the three (Merged). *Scale-bars*: 5 μm. **c** Neutralization efficiency of post-immune serum against CpMEDLE-1 in *C. parvum* culture. Hypochlorite-treated oocysts were pre-incubated in medium with 1:1,000, 1:500 and 1:200 dilutions of pre- and post-immune serum, with medium alone as a control. Levels of infection in the presence of post-immune serum were normalized using data from cultures treated with the pre-immune serum. Data presented are mean ± SD from three replicate assays

To assess the expression profile of the CpMEDLE-1 gene during intracellular development, qPCR was performed over a 72 h time course in *C. parvum*-infected HCT-8 cells. When normalized with data from the *Cp18S* rRNA gene, the expression of CpMEDLE-1 decreased gradually during the culture period (Fig. 3a). The highest expression level was detected at 2 h post-infection.

In examination of CpMEDLE-1 expression by immunofluorescence microscopy of developmental stages, the anti-CpMEDLE-1 antibodies (~0.4 μg/ml) reacted with the mid-anterior region of sporozoites (Fig. 3b, top panel). At 24 h of the cell culture, type I meronts had the highest of reactivity to the antibodies in part of the merozoites (Fig. 3b, middle panel). At 48 h, the reactivity of the antibodies to mature type I meronts appeared to be still restricted to part of the merozoites (Fig. 3b, bottom panel).

### Inhibition of *C. parvum* invasion by anti-CpMEDLE-1 antibodies

Neutralization assays were carried out to assess the effect of anti-CpMEDLE-1 antibodies on *C. parvum* invasion of HCT-8 cells. In comparison with the control culture, the mean parasite loads had a modest but significant reduction when the cell culture was inoculated with sporozoites treated with antiserum from CpMEDLE-1 immunized rabbits (Fig. 3c). The inhibitory effect was 21.9% (26.8 ± 3.5 and 20.9 ± 4.1 per 200× field for pre- and post-immune serum, respectively; *t*_(2)_ = 8.154, *P* = 0.015) at the 1:1000 dilution, 39.0% (25.5 ± 4.2 and 15.6 ± 1.2 per 200× field for pre- and post-immune serum, respectively; *t*_(2)_ = 4.983, *P* = 0.038) at the 1:500 dilution, and 37.0% (24.3 ± 5.2 and 15.3 ± 3.0 per 200× field for pre- and post-immune serum, respectively; *t*_(2)_ = 4.678, *P* = 0.043) at the 1:200 dilution.

## Discussion

Our results suggested that CpMEDLE-1 protein could play a role in invasion or early development of *C. parvum* based on the following evidence: (i) the expression of the CpMEDLE-1 gene peaked at 2 h post-infection, just after the entry of sporozoites into culture cells [18]; (ii) polyclonal antibodies against CpMEDLE-1 resulted in a reduction in parasite infection at approximately 40%. The reduction is moderate since apicomplexan are known to use various strategies for invasion [19]; (iii) CpMEDLE-1 was identified to locate in the mid-anterior region of sporozoites. During sporozoite invasion, the molecules mediating attachment tend to locate apically, whereas those mediating invasion can locate elsewhere on the surface of or within sporozoites [20]. Thus, despite CpMEDLE-1 was not located apically as expected, it could still be involved in early growth and development of *C. parvum*.

Although CpMEDLE-1 is considered to participate in invasion or early development of the pathogen, the subcellular location of CpMEDLE-1 remains unclear. Our data localized CpMEDLE-1 to the mid-anterior region of sporozoites. Even though we were unable to position it precisely to any organelle due to the technique used, the mid-anterior location of the protein is in agreement with the location of dense granules [21]. The expression of CpMEDLE-1 in merozoites appears to be similar, as only a small part of the merozoites reacted with anti-CpMEDLE-1 antibodies. Further studies using immunofluorescence electron microscopy are needed for precise subcellular localization of CpMEDLE-1.

In comparison with CpMEDLE-2, CpMEDLE-1 has shown unique sequence characteristics and a very different expression profile [13]. In direct sequence comparison and phylogenetic analysis, CpMEDLE-2 is divergent from CpMEDLE-1 and other MEDLE proteins. This is also supported by comparisons of protein domains, which have shown the presence of ATP-dependent RNA helicase or histone chaperone domains in CpMEDLE-1 and some other MEDLE proteins, compared with the serine/threonine protein kinase domain in CpMEDLE-2. While the expression of CpMEDLE-1 in sporozoites appears to be restricted to dense granules, the expression of CpMEDLE-2 is diffused throughout the sporozoites. The expression of genes encoding the two proteins also appears to be significantly different *in vitro* culture, with peak expression at 2 h and 48 h, respectively. Thus, data obtained thus far suggest that MEDLE proteins are developmentally regulated and may expert their functions in different developmental stages.

The function of MEDLE proteins is unclear, but here we offer a hypothesis on their role in immune evasion by the *C. parvum*. As predicted *in silico*, MEDLE proteins are characterized with the presence of domains for ATP-dependent RNA helicase, histone chaperone, and serine/threonine protein kinase. The former two are known to be involved in transcriptional regulations while the latter is well known to target immunity-related GTPases (IRGs) for host immunity evasion in *Toxoplasma gondii* [22, 23]. Thus, in addition to regulating parasite gene transcription during parasite growth and development, the secretion of these proteins into host cells during invasion could modulate host gene expression and serve as an immune evasion mechanism. Indeed, recent studies have demonstrated the delivery of several *C. parvum* RNA transcripts into host cells during the infection, modulating the transcription of host genes involved in immune defense [24]. It was further demonstrated that histone modification-mediated epigenetic mechanisms may contribute to this nuclear delivery and transcriptional suppression [25].

The potential role of MEDLE proteins in immune evasion is also supported by the presence of LCRs. LCRs are prevalent in *Cryptosporidium* spp. and other apicomplexans, characterized by low diversity in residues, such as homopolymers, short tandem repeats or aperiodic mosaics [26, 27]. Previous studies had shown that these regions might undergo rapid evolution due to their high compositional plasticity [28], or play a role in immune invasion because of the ability in swiftly changing immunodominant epitopes on antigens [29].

## Conclusions

We have expressed a *Cryptosporidium*-specific secreted protein CpMEDLE-1, and observed significant differences in gene and protein expressions in *C. parvum* compared with the previously studied CpMEDLE-2. Our results indicate that CpMEDLE-1 might be a dense granule protein and potentially could be involved in the transcriptional regulation of the expression of parasite or host proteins. These conclusions should be substantiated with data generated using more advanced molecular biological tools, including those generated using gene knockout approaches such as the CRISPR/Cas9 system.

## Abbreviations

BSA: Bovine serum albumin
DAPI: 4′, 6-diamidino-2-phenylindole
ELISA: Enzyme-linked immunosorbent assay
FBS: Fetal bovine serum
HRP: Horseradish peroxidase
LCRs: Low complexity regions
MALDI-TOF-MS: Matrix-Assisted Laser Desorption/Ionization Time of Flight Mass Spectrometry
PBS: Phosphate-buffered saline
RT: Room temperature
SDS-PAGE: Sodium dodecyl sulfate polyacrylamide gel electrophoresis
